# Finite-stage nonreciprocal thermal circulators for radiative energy harvesting: Benchmarks for isolation, termination, and etendue

**DOI:** 10.1093/pnasnexus/pgag249

**Published:** 2026-07-21

**Authors:** Seungwoo Lee

**Affiliations:** Department of Integrative Energy Engineering, College of Engineering, Korea University, 145 Anam-ro, Seongbuk-gu, Seoul 02841, Republic of Korea; KU-KIST Graduate School of Converging Science and Technology, Korea University, 145 Anam-ro, Seongbuk-gu, Seoul 02841, Republic of Korea

**Keywords:** nonreciprocal thermal radiation, radiative cooling, Landsberg limit, captured etendue, thermal circulators

## Abstract

Reaching the Landsberg limit for radiative energy conversion requires controlling not only spectra but also the direction in which radiative entropy is transported. We develop a finite-stage nonreciprocal thermal-circulator framework for positive illumination (solar conversion) and negative illumination (radiative-cooling work extraction). The ideal theory is expressed in terms of open-system exergy and a minimal port-network picture: a three-port circulator with a cold termination acts as a two-port thermal isolator, and cascaded isolators enforce directed adjacency between effective photon reservoirs. In response to the central practical limitation of nonreciprocal radiative cooling, emphasized recently by Liu et al. we recast the problem as a benchmark map rather than a claim of immediate device-level cooling enhancement. The reported work output values (in unit of W m^−2^) are hemispherical-equivalent upper envelopes; experimentally relevant output is reduced by captured free-space etendue, coupling efficiency, finite isolation ratio, finite termination temperature, atmospheric transmission, and out-of-window loss. We therefore introduce explicit sensitivity factors for isolation, termination, and captured etendue and ask how good a nonreciprocal system must be before the finite-stage advantage remains measurable. Finite-stage calculations give temperature and wavelength targets for 3–5 stages, while the practical benchmark identifies the isolation, termination, and coupling requirements needed to make those targets consequential. This formulation links ideal Landsberg-type limits to testable design requirements for nonreciprocal thermal photonics.

Significance Statement Nonreciprocal thermal photonics promises to route thermal radiation in ways forbidden by reciprocal detailed balance, but recent work has emphasized that realistic nonreciprocal radiative cooling gains can be strongly suppressed by spectral, angular, geometric, and loss constraints. We address this gap by converting finite-stage Landsberg-type limits into a practical benchmark map. The framework quantifies how finite isolation ratio, finite termination temperature, captured free-space etendue, and coupling efficiency reduce the ideal gain. It also clarifies that hemispherical work output values are upper-envelope references unless a coupling architecture is specified. The result is a design-oriented criterion for deciding when nonreciprocal radiative routing matters experimentally.

## Introduction

Radiative energy conversion is constrained by the entropy carried by photons. For positive illumination, solar conversion can be improved by spectral splitting and time-asymmetric routing, but reciprocal detailed balance limits the achievable work in ordinary optical channels (1, 2). For negative illumination, outgoing terrestrial thermal radiation can in principle be used as a work resource, yet the same entropy constraints limit radiative-cooling power generation (3, 4). Nonreciprocal thermal photonics introduces a mechanism for separating resource ports from waste ports, potentially allowing a radiative system to approach Landsberg-type reversible limits (5, 6).

A key unresolved issue is not whether ideal nonreciprocal ladders possess a thermodynamic advantage. It is whether that advantage survives the constraints of real devices. This point was sharpened by the recent commentary by Liu et al. (7), which argued that nonreciprocal thermal radiation is unlikely to improve conventional passive radiative cooling unless stringent spectral selectivity, angular asymmetry, geometric asymmetry, and low out-of-window loss are simultaneously achieved. We agree with this assessment for direct device-level cooling enhancement. Our purpose here is therefore different and complementary: to build a quantitative benchmark that maps ideal finite-stage nonreciprocal limits onto measurable performance under finite isolation, finite termination temperature, and finite captured etendue.

**Figure 1:**
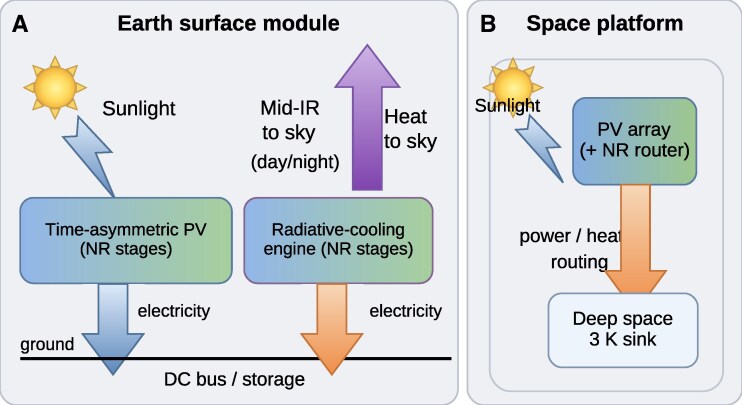
Conceptual platform for positive and negative illumination. The same port-reservoir framework describes solar harvesting and radiative-cooling work extraction. The practical benchmarks developed in the text quantify how much of the ideal finite-stage gain remains after finite isolation, termination, and captured-etendue constraints are applied. A two-panel schematic showing an Earth-surface module and a space platform connected to solar, sky, and electrical ports.

The central question becomes: how good must the nonreciprocal system be before it matters experimentally? We answer this question by combining a compact exergy description with a minimal port-routing model and an explicit sensitivity map. This reframes the finite-stage circulator ladder from an ideal-limit construction into a practical benchmark problem. Figure 1 provides the system-level setting. The same port-reservoir description applies to positive illumination, where solar input must be separated from re-emission, and to negative illumination, where outgoing thermal radiation must be routed to cold sky/space ports while unwanted backflow is terminated. The figure is therefore not a separate platform proposal but an organizing map: it identifies the reservoirs, ports, and electrical outputs to which the later isolation, termination, and captured-etendue benchmarks apply.

## Compact finite-stage framework

The main text keeps only the compact thermodynamic and routing relations needed to interpret the benchmarks; detailed generalized Kirchhoff, BRDF, and modal-radiation derivations are provided in the Supplementary material, where they can be consulted without turning the main article into a review.

### Open-system exergy

For a radiative exchange delivering net energy flux ΔΦ and entropy flux ΔΨ to a device in an environment at temperature $T_{a}$, the maximum reversible work is


(1) $$W_{\max}=\Delta \Phi -T_{a}\Delta \Psi .$$

For sunlight modeled as a blackbody source at $T_{s}$ with geometric concentration factor $\eta_{\mathrm{geo}}$, the full-concentration Landsberg efficiency is


(2) $$\eta_{L}^{\left(+\right)}=1-\frac{4}{3}\frac{T_{a}}{T_{s}}+\frac{1}{3}{\left(\frac{T_{a}}{T_{s}}\right)}^{4},$$

with the corresponding nonfull-concentration expression given in Section S1. For outgoing radiation to an effective sink temperature $T_{o}$, the negative-illumination Landsberg work density is (4)


(3) $$W_{L}^{\left(-\right)}=\frac{1}{3}\sigma T_{a}^{4}-\frac{4}{3}\sigma T_{a}T_{o}^{3}+\sigma T_{o}^{4},$$

where *σ* is the Stefan–Boltzmann constant. The detailed radiation energy and entropy flux identities are summarized in Section S1.

### Finite-stage ladders

In a negative-illumination ladder, $T_{0}=T_{o}<T_{1}<\cdots <T_{N}<T_{N+1}=T_{a}$. Directed routing enforces an adjacency constraint: stage *i* absorbs only from the immediately hotter neighbor and emits only toward the immediately colder neighbor. The finite-stage work is


(4) $$W_{N}^{\left(-\right)}=\sum_{i=1}^{N}\left[\Phi (T_{i})-\Phi (T_{i-1})\right]\left(\frac{T_{a}}{T_{i}}-1\right),$$

where Φ(T) can be full-spectrum or band-limited. For positive illumination, with $T_{s}=T_{0}>T_{1}>\cdots >T_{N}>T_{N+1}=T_{c}$,


(5) $$W_{N}^{\left(+\right)}=\eta_{\mathrm{geo}}\sum_{i=1}^{N}\left[\Phi (T_{i-1})-\Phi (T_{i})\right]\left(1-\frac{T_{c}}{T_{i}}\right).$$

The derivations and numerical optimization methods are given in Sections S1–S3.

### Minimal routing primitive

**Figure 2:**
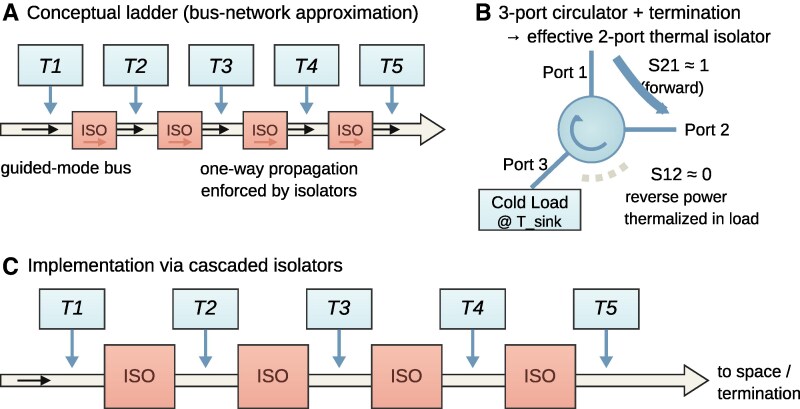
Directed routing primitive. A) Conceptual directed ladder. B) A three-port circulator terminated in a cold load acts as an effective thermal isolator. C) Cascaded isolators realize directional adjacency along a guided-mode bus. The termination is a physical cold waste port at $T_{\mathrm{sink}}$, not a bookkeeping device. Three schematics showing a ladder, a circulator with a cold load, and a cascaded isolator bus.

The finite-stage equations require optical adjacency that ordinary reciprocal radiation does not provide. Figure 2 addresses this implementation problem. Figure 2(a) shows the directed ladder as a sequence of effective photon reservoirs. Figure 2(b) presents the minimal nonreciprocal primitive: a three-port circulator terminated at one port forms an effective two-port thermal isolator. Reverse power is not destroyed; it is redirected into a cold load at $T_{\mathrm{sink}}$, which must be sufficiently cold that its thermal noise does not re-enter the resource pathway. Figure 2(c) shows the corresponding guided-mode implementation by cascaded isolators. This figure is essential because it converts the abstract ladder constraint into a device-level requirement: useful nonreciprocity requires both high forward transmission and a real, thermally anchored waste port.

Only the consequences of this port framework are needed in the main text. The generalized Kirchhoff relations, BRDF identities, row/column defect relations, and connection to universal modal radiation laws are collected in Section S4 (6, 8–10). Moving these formal relations to the Supplementary material keeps the main manuscript focused on the experimentally relevant benchmark: the amount of ideal finite-stage gain that remains after finite isolation, finite termination temperature, and captured etendue are included.

**Figure 3:**
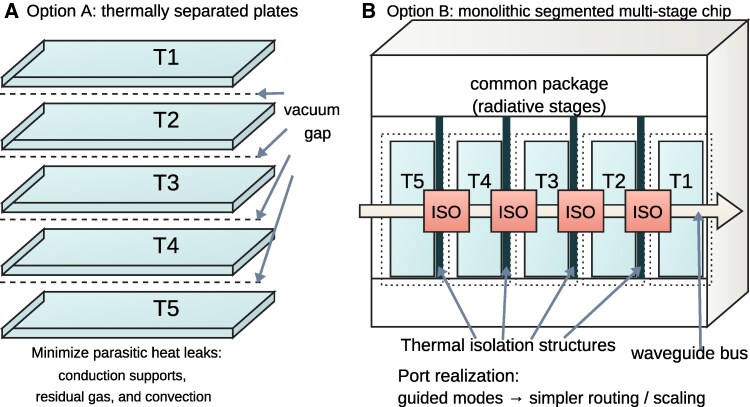
Two hardware interpretations of a finite-stage nonreciprocal ladder. A) Thermally separated plates expose vacuum-gap and support-related parasitics. B) A monolithic guided-mode implementation uses suspended membranes, indicated by dotted outlines, to isolate stages while routing radiation through a nonreciprocal bus. The comparison identifies the dominant practical loss channels that enter the benchmark factors in Table 1. Two schematic device architectures showing separated plates and a monolithic suspended-membrane guided-mode chip.

Figure 3 compares two hardware interpretations. Option A uses thermally separated plates and makes parasitic conduction, residual gas conduction, and convection explicit. Option B implements the same ladder as a monolithic suspended-membrane guided-mode network, with dotted outlines indicating membranes thermally isolated from the substrate. The figure should be read as a map of failure channels: Option A exposes macroscopic heat leaks, whereas Option B exposes finite isolation, guided-mode coupling, and termination design as the dominant bottlenecks. This interpretation connects directly to the practical sensitivity factors introduced below.

## Practical benchmark map

The ideal finite-stage works in Eqs. 4 and 5 are upper-envelope quantities. To compare with real systems, three additional reductions must be applied: the fraction of free-space etendue captured by the engineered port set, the coupling efficiency into that port set, and the quality of the nonreciprocal routing/termination. We write the negative-illumination areal output as


(6) $$W_{\mathrm{area}}^{\left(-\right)}\approx (f_{\Omega }\eta_{\mathrm{cpl}})\;\eta_{\mathrm{iso}}\;\eta_{\mathrm{term}}\;W_{N,\mathrm{hemi}}^{\left(-\right)},$$

where $W_{N,\mathrm{hemi}}^{\left(-\right)}$ is the hemispherical-equivalent value reported by the ideal calculation, $f_{\Omega }$ is the captured free-space etendue fraction, $\eta_{\mathrm{cpl}}$ is the port-coupling efficiency, $\eta_{\mathrm{iso}}$ accounts for finite isolation ratio, and $\eta_{\mathrm{term}}$ accounts for the finite temperature of the termination load. For positive illumination, the corresponding retained nonreciprocal correction is


(7) $$\eta_{\mathrm{eff}}^{\left(+\right)}\approx \eta_{\mathrm{MC}}+\eta_{\mathrm{iso}}\eta_{\mathrm{term}}\left(\eta_{N}^{\left(+\right)}-\eta_{\mathrm{MC}}\right),$$

with $\eta_{\mathrm{MC}}$ the reciprocal multicolor reference. These relations are not a replacement for full device simulation. They are first-pass benchmark equations that answer the central practical question: how good must the nonreciprocal system be before the ideal gain remains observable?

Table 1 summarizes the practical interpretation. For example, the often-quoted 22.7 W m^−2^ window-selective finite-stage value at $T_{a}=300$ K is not a direct output of a single guided-mode bus. If $f_{\Omega }\eta_{\mathrm{cpl}}=10^{-2}$, the same thermodynamic benchmark corresponds to an areal contribution of order 0.23 W m^−2^ before isolation and termination penalties. Conversely, dense parallel arrays or metasurface/grating couplers that map many angular channels into engineered ports could increase $f_{\Omega }\eta_{\mathrm{cpl}}$. This is a coupling-engineering requirement, not a change in the thermodynamic bound.

**Table 1.** Practical sensitivity benchmark.

Quantity	Screening criterion	Interpretation
Isolation ratio	I≳30 –40 dB for a 3–5 stage ladder	Below ∼20 dB, accumulated reverse leakage can suppress most of the finite-stage correction.
Termination temperature	$T_{\mathrm{sink}}$ should be near the cold reservoir coupled to the waste port	A warm termination re-injects thermal noise and reduces $\eta_{\mathrm{term}}$; the ideal cold-load assumption is a stringent design requirement.
Captured etendue	Report both $W_{N,\mathrm{hemi}}$ and $(f_{\Omega }\eta_{\mathrm{cpl}})W_{N,\mathrm{hemi}}$	Hemispherical W m^−2^ numbers are upper envelopes unless free-space coupling is specified.
Atmospheric window	Couple primarily through transparent sky/space bands	The Joule analysis shows that nonatmospheric-window loss can overwhelm any nonreciprocal cooling benefit.

Values are intended as design filters rather than final device predictions. *I* is an isolation ratio, $\epsilon =10^{-I/10}$ is the reverse leakage fraction, and $f_{\Omega }\eta_{\mathrm{cpl}}$ maps the hemispherical-equivalent benchmark to a captured-port areal output.

This perspective also clarifies the relation to Liu et al. (7). Their commentary argues that nonreciprocal thermal radiation is unlikely to improve conventional radiative-cooling efficiency unless nonreciprocity is combined with strong spectral selectivity, angular/geometric asymmetry, and suppression of loss outside atmospheric windows. Our benchmark is consistent with that conclusion. It does not claim that nonreciprocity alone produces large outdoor cooling gains. Instead, it quantifies how finite isolation, termination, and captured etendue reduce the ideal finite-stage advantage, and therefore identifies the operating window in which a nonreciprocal thermal circulator could be experimentally meaningful.

## Case study I: negative illumination

We first consider radiative-cooling work extraction. The ideal emitter models are: (i) a full-spectrum blackbody reference, (ii) an ideal atmospheric-window emitter with ϵ=1 in 7–15 μm and zero elsewhere, and (iii) an ideal broadband Earth emitter with out-of-window channels coupled back to the ambient atmosphere. The stage temperatures are optimized subject to $260\;K\leq T_{i}\leq T_{a}$.

**Figure 4:**
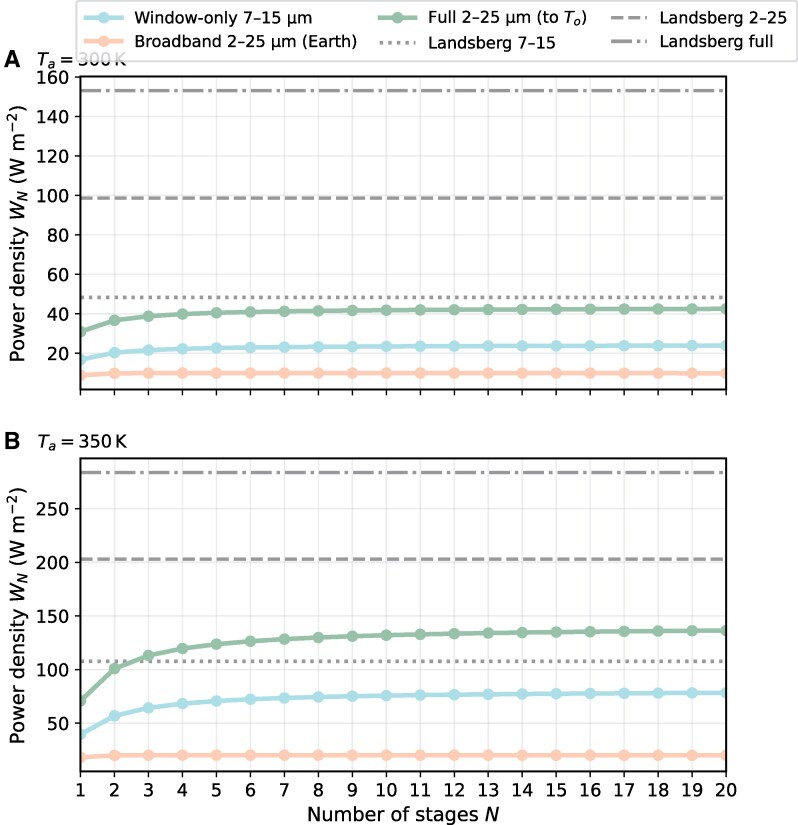
Finite-stage negative-illumination work benchmarks. A) $T_{a}=300$ K. B) $T_{a}=350$ K. The plotted W m^−2^ values are hemispherical-equivalent upper envelopes. Two line plots showing generated work density versus number of nonreciprocal stages for several spectral scenarios.

Figure 4 shows the finite-stage work for $T_{a}=300$ K and $T_{a}=350$ K. Each point is a separate constrained optimization. The window-selective curve exceeds the broadband-Earth curve because out-of-window atmospheric coupling behaves as a heat leak when the stages are sub-ambient. The full-spectrum blackbody curve is an upper reference rather than an Earth-realizable outdoor prediction. The two panels should be interpreted through Eq. 6: the plotted values are hemispherical-equivalent benchmarks. Real areal output requires multiplying by captured etendue, coupling efficiency, isolation quality, and termination quality. The elevated $T_{a}=350$ K case is included because solar-loaded emitters and photovoltaic modules often operate above 300 K; it tests whether the same nonreciprocal architecture remains beneficial under hotter operating conditions.

**Figure 5:**
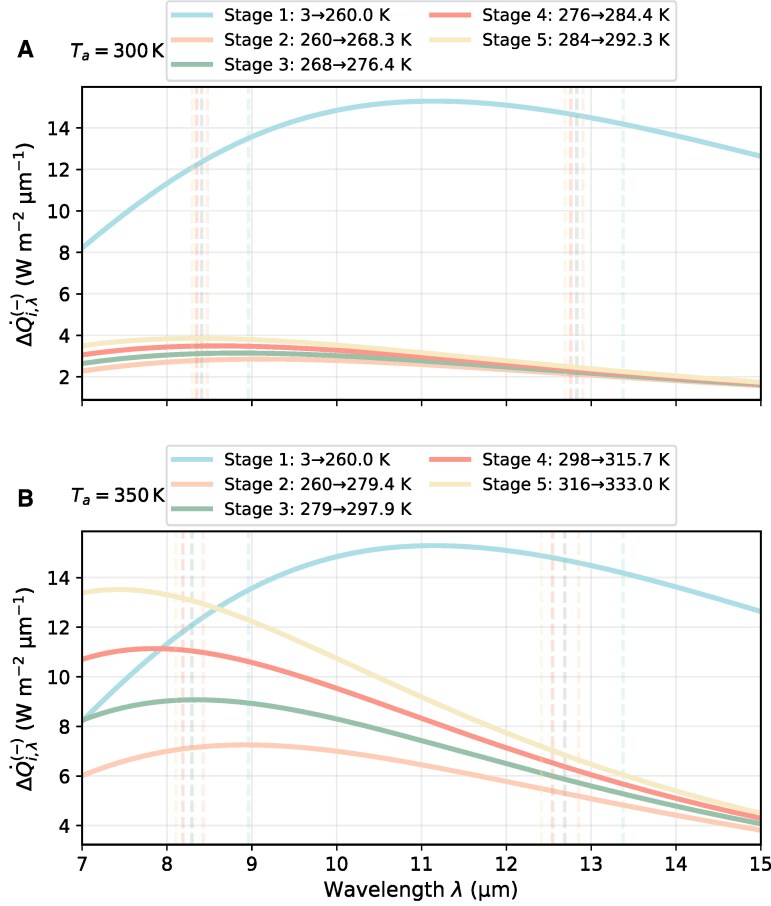
Stage-resolved target bands for negative illumination. The curves show incremental spectral heat fluxes and the vertical markers indicate 20–80% contribution windows. Two spectral plots of mid-infrared heat-flux contributions for different stages.

Figure 5 resolves the incremental spectral heat fluxes for the 7–15 μm window case. The 20–80% contribution bands define where a nonreciprocal isolator or resonant routing element must provide strong directionality. The hottest stages shift the target bands toward shorter wavelengths, but the dominant design region remains inside the atmospheric window. This figure is the spectral counterpart of the practical benchmark: even if a system has excellent isolation, it matters only in the modes that carry most of the incremental heat flux.

## Case study II: positive illumination

We next consider the time-reversed solar problem, using $T_{s}=6,000$ K, full concentration, and cell temperatures $T_{c}=300$ and 350 K. The reciprocal multicolor limit is used as the baseline; the finite-stage nonreciprocal correction is evaluated with Eq. 5. This setting connects directly to time-asymmetric photovoltaic proposals (1, 2) and to related ideas in thermophotovoltaics (11).

**Figure 6:**
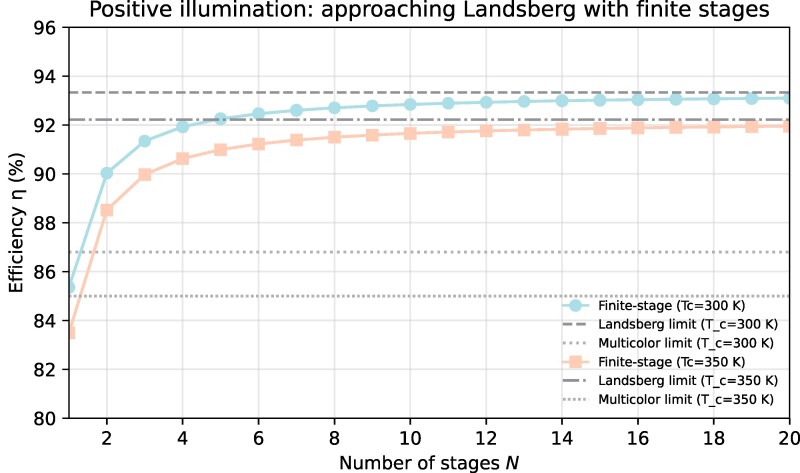
Finite-stage positive-illumination efficiency. The curves show the approach to Landsberg-like performance for $T_{c}=300$ and 350 K, compared with reciprocal multicolor baselines. Line plot of solar conversion efficiency versus number of nonreciprocal stages.

Figure 6 shows that a few stages recover most of the ideal gap between the reciprocal multicolor limit and the Landsberg limit. The saturation with *N* is important: once 3–5 stages are present, further improvement depends more on port quality than on adding more ideal stages. Equation 7 therefore provides the practical reading of the figure: if isolation or termination quality is poor, the nonreciprocal correction collapses back toward the reciprocal multicolor baseline.

**Figure 7:**
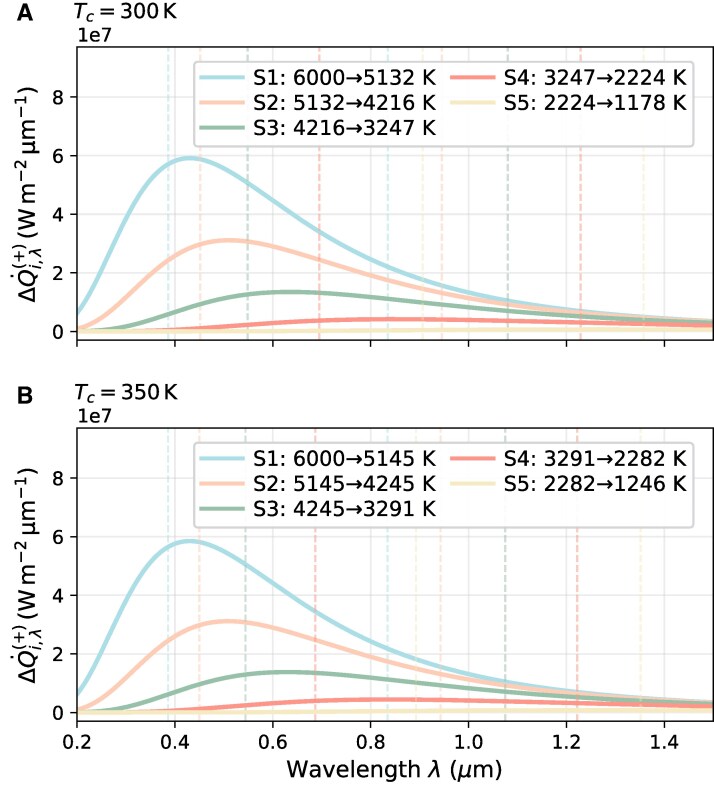
Stage-resolved target bands for positive illumination. The bands indicate where nonreciprocal routing must be effective for each finite stage. Two wavelength plots showing visible and near-infrared spectral contributions for solar conversion stages.

Figure 7 gives the corresponding positive-illumination target bands. The target wavelengths move from visible/near-infrared bands for the hottest stages toward longer wavelengths for the lowest-temperature stages. These bands do not require literal blackbody radiators at thousands of kelvin; they represent effective photon reservoirs or biased luminescent channels in a time-asymmetric converter. As in the negative-illumination case, the spectral target map tells experimentalists where isolation and termination quality are actually needed.

## Discussion

The main conclusion is that finite-stage nonreciprocal thermal circulators are useful as a benchmark only when their port imperfections are made explicit. The ideal ladder calculation answers what is thermodynamically possible, but the practical benchmark map answers what is experimentally consequential.

The commentary by Liu et al. (7) places an important constraint on this interpretation: nonreciprocal radiative cooling does not automatically outperform reciprocal cooling, especially when atmospheric absorption, nonatmospheric-window loss, geometric symmetry, and imperfect angular response are included. Our results should therefore be read as a complementary benchmark for staged work extraction and port-routing quality, not as a stand-alone claim of large passive cooling enhancement. The same conclusion follows from the captured-etendue factor in Eq. 6. Guided-mode ports capture only a small fraction of the hemispherical free-space channel set unless supported by large-area coupling, angular multiplexing, or dense arrays. Reporting both hemispherical-equivalent and per-captured-etendue performance is therefore essential.

From a design standpoint, the most important requirements are: high forward transmission, reverse isolation above roughly 30–40 dB across the working bands, cold and well-anchored termination ports, selective coupling to atmospheric-window modes for radiative cooling, and scalable free-space-to-port coupling. Photonic crystals, ENZ structures, coherent/directional thermal emitters, gratings, and metasurfaces can provide the spectral and angular selectivity needed to define useful ports (12–17); nonreciprocal media or modulation are required to route those ports directionally. These design directions connect to third-generation photovoltaic concepts (18) and are supported by recent experimental demonstrations of Kirchhoff-law violation and strong nonreciprocal thermal emission (19, 20).

## Materials and methods

Band-limited blackbody fluxes were evaluated by numerical quadrature over wavelength. Negative-illumination temperature sequences were optimized by dynamic programming over a monotonic temperature grid, and positive-illumination sequences were optimized by constrained nonlinear optimization. Stage-resolved target bands were defined as the 20–80% cumulative-contribution interval of the incremental spectral heat-flux distribution. Details are given in Sections S1–S3. Generalized Kirchhoff relations, BRDF identities, and modal-radiation framework are further detailed in Section S4. The practical sensitivity factors in Table 1 are first-order screening relations derived in Section S5.

## Supplementary Material

pgag249_Supplementary_Data

## Data Availability

The numerical results were generated from the equations and procedures described in the main text and Supplementary material. The code used to generate the numerical results and figures is available in a public repository: https://github.com/NEOlab-code/nonreciprocal-DB.
