# Supplementary material for: Finite-stage nonreciprocal thermal circulators for radiative energy harvesting: Benchmarks for isolation, termination, and etendue
Source: PNAS Nexus. 2026 Jul 21;5(7):pgag249. doi: 10.1093/pnasnexus/pgag249 (PMC13419078; doi:10.1093/pnasnexus/pgag249)
Supplement: pgag249_Supplementary_Data [file pgag249_supplementary_data.pdf]

# Supporting Information for: Finite-Stage Nonreciprocal Thermal Circulators for Radiative Energy Harvesting: Benchmarks for Isolation, Termination, and Etendue

Seungwoo Lee[1,2]

[1]Department of Integrative Energy Engineering, College of Engineering, Korea University, 145 Anam-ro, Seongbuk-gu, Seoul 02841, Republic of Korea

[2]KU-KIST Graduate School of Converging Science and Technology, Korea University, 145 Anam-ro, Seongbuk-gu, Seoul 02841, Republic of Korea

Corresponding author: seungwoo@korea.ac.kr

## S1 Radiative flux identities

A blackbody radiation field has hemispherical spectral exitance

$$M_\lambda(T) = \frac{2\pi hc^2}{\lambda^5}\left[\exp\left(\frac{hc}{\lambda k_B T}\right) - 1\right]^{-1}. \tag{S1}$$

The energy and entropy fluxes used in the main text are

$$\Phi(T) = \int \epsilon(\lambda) M_\lambda(T)\, d\lambda, \tag{S2}$$

$$\Psi(T) = \int \epsilon(\lambda) S_\lambda(T)\, d\lambda, \tag{S3}$$

with $\Psi = (4/3)\sigma T^3$ for a full blackbody. These relations give the open-system exergy $W = \Delta\Phi - T_a \Delta\Psi$.

## S2 Band-limited work and target-band construction

For a band $[\lambda_1, \lambda_2]$, the band-limited fluxes are obtained by restricting the integral to the band. The negative-illumination incremental spectrum between stages $T_{i-1}$ and $T_i$ is

$$\Delta\dot{Q}^{(-)}_{i,\lambda} = \epsilon(\lambda)\left[M_\lambda(T_i) - M_\lambda(T_{i-1})\right]. \tag{S4}$$

The positive-illumination analogue is

$$\Delta\dot{Q}^{(+)}_{i,\lambda} = M_\lambda(T_{i-1}) - M_\lambda(T_i). \tag{S5}$$

The 20–80% design band is obtained from the cumulative integral of $\Delta\dot{Q}_{i,\lambda}$.

## S3 Optimization procedure

Negative-illumination stage temperatures are optimized by dynamic programming over monotonic temperature grids. Positive-illumination stage temperatures are optimized with constrained nonlinear optimization subject to $T_s > T_1 > \cdots > T_N > T_c$. All curves in the main text are regenerated from these equations. The code package, which is available at https://github.com/NEOlab-code/nonreciprocal-DB, contains the numerical routines and scripts.

## S4 Generalized Kirchhoff, BRDF, and modal-radiation framework

The main text intentionally avoids a long pedagogical derivation of generalized Kirchhoff relations. The formal background is collected here to preserve technical completeness while keeping the main article focused on benchmark consequences.

For an opaque surface with bidirectional reflectance distribution function (BRDF) $\rho(\hat{n}_i \to \hat{n}_j)$, equilibrium arguments give

$$e(\hat{n}_j) + \int \rho(\hat{n}_i \to \hat{n}_j) d\Omega_i = 1, \tag{S6}$$

$$\alpha(-\hat{n}_j) + \int \rho(\hat{n}_j \to \hat{n}_i) d\Omega_i = 1. \tag{S7}$$

Thus

$$\alpha(-\hat{n}_j) - e(\hat{n}_j) = \int \left[\rho(\hat{n}_i \to \hat{n}_j) - \rho(\hat{n}_j \to \hat{n}_i)\right] d\Omega_i. \tag{S8}$$

Reciprocity makes the integrand vanish pairwise; nonreciprocity permits port-level differences while preserving equilibrium when all ports and internal fluctuations are included.

For a multiport scattering matrix $S(\omega)$,

$$A_j(\omega) = 1 - \sum_i |S_{ij}(\omega)|^2, \tag{S9}$$

$$E_i(\omega) = 1 - \sum_j |S_{ij}(\omega)|^2. \tag{S10}$$

Column defects describe absorption from incident ports; row defects describe emission into outgoing ports. In a reciprocal basis the corresponding port pairs coincide, whereas in nonreciprocal structures row and column defects can differ. This is the finite-dimensional port version of the universal modal radiation laws, which state that emission and absorption constraints are properly paired in a modal basis rather than in arbitrary projected ports.

## S5 Practical sensitivity analysis

This section expands the practical benchmark map introduced in the main text. The purpose is to quantify, at a screening level, how isolation ratio, termination temperature, and captured etendue reduce the ideal finite-stage limits.

### S5.1 Finite isolation ratio

Let $I$ be the isolation ratio in dB and $\epsilon = 10^{-I/10}$ the reverse leakage fraction. For an $N$-stage ladder, a conservative first-order retained fraction is

$$\eta_{\text{iso}} \simeq \max\left[0, 1 - N\chi\epsilon\right], \tag{S11}$$

where $\chi$ is an order-unity penalty depending on whether leaked photons return to a resource channel or are thermalized in a waste load. For $N = 5$, isolation of 10 dB can remove a large fraction of the finite-stage correction, whereas 30–40 dB makes leakage a perturbation. The corresponding illustrative sensitivity factors for a 5-stage ladder, evaluated with $\chi = 1$, are summarized in Table S1.

Table S1: Illustrative sensitivity factors for a 5-stage ladder, assuming $\chi = 1$.

| Isolation $I$ | $\epsilon = 10^{-I/10}$ | First-order retained fraction $1 - 5\epsilon$ |
|---|---|---|
| 10 dB | 0.10 | 0.50 |
| 20 dB | 0.01 | 0.95 |
| 30 dB | 0.001 | 0.995 |
| 40 dB | 0.0001 | 0.9995 |

## S5.2 Finite termination temperature

A termination at $T_{\text{sink}}$ emits thermal noise into the load channel. A useful screening factor is

$$\eta_{\text{term}} \simeq 1 - \frac{\Phi(T_{\text{sink}})}{\Phi(T_{\text{stage}})}, \tag{S12}$$

with the band-limited $\Phi$ appropriate to the terminated channel. The penalty is small only if the termination is colder than the relevant stage in the active band and well isolated from the hot package.

## S5.3 Captured etendue and areal scaling

The hemispherical-equivalent value $W_{N,\text{hemi}}$ assumes that all relevant free-space radiance channels are mapped into the directed ladder. A real guided-mode device captures a fraction

$$f_\Omega \eta_{\text{cpl}} = \frac{G_{\text{captured}}}{G_{\text{hemisphere}}} \eta_{\text{cpl}}, \tag{S13}$$

where $G$ is etendue. Therefore

$$W_{\text{area}} \approx (f_\Omega \eta_{\text{cpl}}) \eta_{\text{iso}} \eta_{\text{term}} W_{N,\text{hemi}}. \tag{S14}$$

This is why per-mode or per-captured-etendue reporting is often more transferable than raw hemispherical W $\text{m}^{-2}$ values.

## S5.4 Relation to atmospheric radiative cooling

The commentary by Liu *et al.* (*Joule*, 9, 101887, 2025) emphasizes that nonreciprocity alone is not sufficient to improve passive radiative cooling: the emitter must also avoid losses outside atmospheric windows, maintain the required angular/geometric asymmetry, and operate under realistic sky radiance. The benchmark equations above are consistent with that conclusion. They quantify how quickly the ideal finite-stage gain is reduced once captured etendue, isolation, termination, and atmosphere-aware losses are included.
